# Full-Genome Assembly of Reference Strain *Providencia stuartii* ATCC 33672

**DOI:** 10.1128/genomeA.01082-14

**Published:** 2014-10-23

**Authors:** K. G. Frey, K. A. Bishop-Lilly, H. E. Daligault, K. W. Davenport, D. C. Bruce, P. S. Chain, S. R. Coyne, O. Chertkov, T. Freitas, J. Jaissle, G. I. Koroleva, J. T. Ladner, T. D. Minogue, G. F. Palacios, C. L. Redden, Y. Xu, S. L. Johnson

**Affiliations:** a Naval Medical Research Center (NMRC), Frederick, Fort Detrick, Maryland, USA; b Henry M. Jackson Foundation, Bethesda, Maryland, USA; c Los Alamos National Laboratory (LANL), Los Alamos, New Mexico, USA; d Diagnostic Systems Division (DSD), United States Army Medical Research Institute of Infectious Diseases (USAMRIID), Fort Detrick, Maryland, USA; e Center for Genome Sciences (CGS), USAMRIID, Fort Detrick, Maryland, USA

## Abstract

A member of the normal human gut microflora, *Providencia stuartii*, is of clinical interest due to its role in nosocomial infections of the urinary tract and because it readily acquires antibiotic resistance. Here, we present the complete genome of *P. stuartii* strain ATCC 33672, consisting of a 4.28-Mbp chromosome and a 48.9-kbp plasmid.

## GENOME ANNOUNCEMENT

*Providencia stuartii* is a frequent cause of urinary tract infections in hospital patients with long-term indwelling catheters (1). In these patients, bacteriuria may rapidly progress to bacteremia (2, 3). Infection with *P. stuartii* has also been reported to cause peritonitis (4), meningitis (5), pericarditis (6), and infective endocarditis (7). In an unusual case, *P. stuartii* was identified as the cause of a renal abscess mistakenly attributed to an infection with *Pasteurella* (8). Additionally, nosocomial dissemination of multidrug-resistant *P. stuartii* has been reported in burn units (9, 10) and an intensive care unit (10). Members of the genus *Providencia* harbor resistance to aminopenicillins and narrow-spectrum cephalosporins (11) but also readily acquire resistance to other antibiotics through horizontal transfer (12, 13). This is of great concern to public health due to the ability of *P. stuartii* to further disseminate antimicrobial resistance genes (13, 14). We present the complete genome of strain ATCC 33672, consisting of a 4.28-Mbp chromosome and a 48.9-kbp plasmid.

High-quality genomic DNA was extracted from a purified isolate using the Qiagen Genome-tip 500. Specifically, a 100-ml bacterial culture was grown to stationary phase and nucleic acid extracted as per the manufacturer’s recommendations. The draft genome of *P. stuartii* ATCC 33672 included a combination of Illumina (15) and 454 technologies (16). For this genome, we constructed and sequenced a 100-bp Illumina library (372-fold genome coverage) and a long-insert paired-end 454 library (6,689 ± 1,140-bp insert; 5-fold genome coverage). Raw data are available in the Short Read Archive (SRA) under accession numbers SRX687103 (Illumina), SRX687104 (Illumina), and SRX687264 (454).

The 454 paired-end data were assembled in Newbler (16), and those consensus sequences were computationally shredded into 2-kbp overlapping fake reads (shreds). The Illumina sequencing data were assembled with Velvet (17), and the consensus sequences were computationally shredded into 1.5-kbp overlapping shreds. All data were additionally assembled in AllPaths (18), and the consensus sequences were computationally shredded into 5-kbp overlapping shreds. We integrated the 454 Newbler consensus shreds, the Illumina Velvet consensus shreds, AllPaths consensus shreds, and the 454 paired-end library read pairs using parallel Phrap (High Performance Software, LLC). Possible misassemblies were corrected and repeat regions verified using in-house scripts and manual editing in Consed (19–21).

The complete genome assembly of *P. stuartii* ATCC 33672, including a 4,285,951-bp circular chromosome (41.5% G+C content) and a 48,866-bp circular plasmid (42.8% G+C content), was annotated utilizing an Ergatis-based (22) workflow with minor manual curation. The annotated genome contains 4,094 predicted genes, including 3,926 protein-coding genes, 80 tRNA genes, and 22 rRNA genes. A total of 92 virulence genes were noted, including resistance to fluoroquinolones, β-lactams, and aminoglycosides.

### Nucleotide sequence accession numbers.

The nucleotide sequences for *P. stuartii* ATCC 33672 have been deposited in GenBank under accession numbers CP008920 (chromosome) and CP008919 (plasmid).
